# Pilot Study Evaluating the Early Clinical Outcomes Obtained with a Novel, Customized, Multifocal Corneo-Scleral Contact Lens for Presbyopia Correction

**DOI:** 10.3390/life15050700

**Published:** 2025-04-25

**Authors:** Laura Barberán-Bernardos, Daniel Soriano Salcedo, Sergio Díaz-Gómez, David P. Piñero

**Affiliations:** 1 Department of Optics, Pharmacology and Anatomy, University of Alicante, 03690 Alicante, Spain; laura.barberan@ua.es; 2 Centro Oftalmológico Integral Bilbao Berri SL, Miranza COI Bilbao, 48008 Bilbao, Spain; danielsoriano1989@gmail.com (D.S.S.); sergio.diaz@miranza.es (S.D.-G.); 3 Department of Ophthalmology (IMQO-Oftalmar), Vithas Medimar International Hospital, 03016 Alicante, Spain

**Keywords:** corneo-scleral contact lens, multifocal contact lens, defocus curve, contrast sensitivity, ocular high-order aberrations

## Abstract

Background: The objective was to preliminarily evaluate the short-term clinical outcomes obtained in presbyopic patients with a novel, multifocal, customized corneo-scleral contact lens (CSCL). Methods: A total of 11 presbyopic subjects (age 45–80 years, corrected-distance visual acuity ≤ 0.1 LogMAR, near addition ≥ +1.00 D) were recruited and fitted with a multifocal corneo-scleral contact lens in this pilot study. Pre-fitting evaluations included stereopsis, contrast sensitivity (CS), and ocular aberrometry, with follow-up assessments conducted at 20 min and 1-month post-fitting. The defocus curve was also measured to assess visual performance across varying distances. Results: Twenty-two eyes from 11 participants (53.9 ± 4.7 years, 10 female) were included in this study. Significant changes were observed post-fitting for primary and secondary spherical aberration, coma, and stereopsis (*p* ≤ 0.033). No significant changes in Strehl ratio and total root mean square were detected (*p* ≥ 0.182). Binocular contrast sensitivity was better with spectacles than with the fitted CSCL at all frequencies (*p* ≤ 0.048), but the change in monocular did not reach statistical significance for 18 cycles per degree (*p* = 0.109). All patients and 90.9% of patients achieved a visual acuity of 0.0 LogMAR or better at distance and at intermediate, respectively, and 91.8% achieved 0.3 LogMAR or better for near vision. Conclusions: The customized CSCL evaluated provided functional recovery of visual quality across distances, with acceptable reductions of CS and stereopsis that are comparable to those reported for other multifocal contact lenses.

## 1. Introduction

Presbyopia is an age-related condition characterized by a gradual reduction in the lens’ accommodative capacity, leading to a progressive decline in near vision [1]. Symptoms typically become noticeable and worsen from the fourth decade of life, affecting approximately 1.8 billion people worldwide [1]. Given the global trend of population aging, the demand for effective presbyopia solutions is expected to rise significantly in the coming years.

The correction of presbyopia with contact lenses was first documented in the 1960s using bifocal designs [2]. However, it was not until the 1980s that the first generation of multifocal contact lenses emerged, demonstrating clinically satisfactory outcomes [3]. Today, numerous optical designs are commercially available in both soft hydrogel and rigid gas-permeable (RGP) materials, employing various optical principles [4]. While extensive research has evaluated the performance of soft multifocal contact lenses, published data on the clinical efficacy of RGP multifocal designs remains limited [5]. It is important to note that multifocal rigid gas permeable (RGP) contact lenses are available in three distinct fitting designs: fully corneal, corneo-scleral, and fully scleral. While these designs may demonstrate comparable optical performance, previous experience suggests that lenses with scleral bearing typically provide superior comfort due to their reduced interaction with the sensitive corneal nerve endings [6].

Proper centration of multifocal contact lenses (CLs) is critical to prevent ghost images and maintain optimal optical quality. Lens movement during blinking or slight decentration can induce aberrations that compromise visual performance [7]. In this context, scleral contact lenses (SCLs) offer advantages by ensuring stable centration while preserving the superior optical quality of rigid gas-permeable (RGP) materials. However, evidence supporting their efficacy remains limited. Available studies have primarily evaluated miniscleral designs, with no published data on multifocal corneo-scleral contact lenses (CSCLs). Privado-Aroco et al. [8] demonstrated that multifocal miniscleral CLs provide enhanced intermediate and near vision while maintaining good distance acuity compared to monofocal designs. Additionally, SCLs with a customized, decentered optical zone have shown superior visual quality over conventional designs [9].

The tear film meniscus can significantly modulate visual quality and depth of focus in multifocal contact lens (CL) wear, with effects ranging from beneficial to detrimental depending on its optical properties [10]. Furthermore, the eye’s inherent higher-order aberrations (HOAs)—particularly primary spherical aberration—critically influence multifocal CL performance since these lenses fundamentally work by inducing controlled amounts of HOAs [11,12]. This interaction between natural ocular aberrations and designed optical profiles underscores the importance of personalized fitting approaches. Thus, optimizing lens design to account for fitting dynamics and ocular optical properties is essential. A fully customized approach is recommended to maximize outcomes. Recently, the Presbycustom lens (Lenticon, Madrid, Spain) became the first commercially available multifocal CSCL for the compensation of presbyopia to incorporate customization based on primary and secondary spherical aberration profiles, compensating also for tear film meniscus effects. This innovation represents a significant advancement in personalized presbyopia correction.

This pilot study aimed to conduct a preliminary evaluation of the short-term clinical performance of a novel, customized, multifocal corneo-scleral contact lens (CSCL) in presbyopic patients, assessing both its clinical viability and potential efficacy. A one-month follow-up period was selected based on established neuroadaptation timelines, as demonstrated by functional magnetic resonance imaging studies showing that primary neural adaptations to new optical corrections typically stabilize within this timeframe [13,14].

## 2. Materials and Methods

### 2.1. Participants

This pilot study was conducted at Centro Oftalmológico Integral Bilbao Berri in Bilbao. All subjects signed an informed consent after they were informed of the details of the study. Ethical approval was obtained from the Ethics Committee of the Health Department of Alicante General Hospital (PI2020-084, ISABIAL 200071), and this study was conducted following the tenets of the Declaration of Helsinki. 

Inclusion criteria were patients between the ages of 45 and 80 years with corrected-distance visual acuity of 0.1 LogMAR or better and a near addition value equal to or greater than 1 D. Additionally, subjects who presented any systemic pathology affecting the ocular surface, active ocular pathologies, or amblyopia with a difference in visual acuity between both eyes in two or more lines were excluded.

### 2.2. Pre-Fitting Examination

All participants underwent a complete visual examination by the same experienced optometrist (D.S.S.) before the fitting of the corneo-scleral contact lenses. This examination included objective refraction with retinoscopy, subjective refraction, a cover test, pupillometry, slit lamp biomicroscopy, corneal topography, ocular aberrometry, pachymetry, measurement of stereopsis, and measurement of monocular and binocular CS. The OPD-Scan III platform (Nidek Technologies, Gamagori, Japan) was used to evaluate corneal topography, ocular aberrometry, and pachymetry. Photopic binocular and monocular (dominant eye) contrast sensitivity for frequencies of 1.5, 6, 12, and 18 cycles/degree was assessed using the validated application ClinicCSF [14]. OptoTab (Smarthin4vision, Zaragoza, Spain) was used to evaluate stereopsis. With this data, the parameters of the first trial multifocal CSCL were calculated following the manufacturer’s guidelines.

### 2.3. Contact Lens Design and Fitting

**Figure 1:**
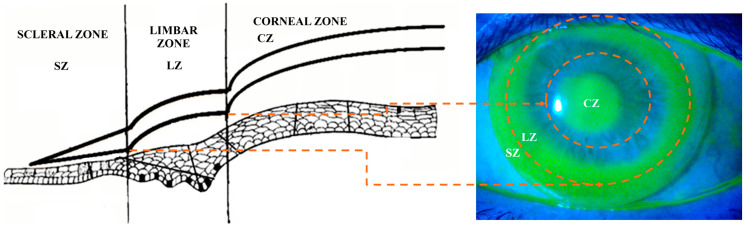
Ideal fluorogram of the corneo-scleral contact lens evaluated (**right**) and diagram of the different parts of the corneo-scleral contact lens (CSCL) (**left**): scleral (SZ), limbal (LZ), and corneal zone (CZ). The orange arrows and circles delimit the transition areas between zones.

The Presbycustom contact lens (Roflufocon D, Dk 125 Fatt units, Contamac, Saffron Wilden, UK) is the result of a proof-of-concept research project developed by the Group of Optics and Visual Perception of the University of Alicante and licensed to Laboratorios Lenticon SA (Madrid, Spain) [15]. It is a corneo-scleral contact lens made of highly gas-permeable material and comprises three different areas: corneal, limbal, and scleral (Figure 1). This allows for optimal fitting of the CSCL to the different areas of the ocular surface, with alignment in the transition zone between the corneal and limbal zones, as shown in Figure 1.

The optics of the lens consist of a 4 mm optical zone modifiable to 3.5 mm, including two additional modalities (low or high) to provide less or greater depth of focus, respectively, and a customizable positive or negative spherical aberration induction depending on the patient’s ocular spherical aberration. The back surface asphericity of the CSCL is fixed, whereas the central anterior surface asphericity is modified according to the aberrometric induction required. Specifically, the depth of focus achieved with the contact lens is set by customizing the induction of primary and secondary spherical aberration. It has been demonstrated that combining primary and secondary spherical aberrations of opposite signs is significantly more effective for expanding the depth of focus than other aberrometric options [16,17]. The scleral area of the contact lens allows for a smooth bearing over the sclero-conjunctival surface, facilitating comfortable wearing.

The optics were customized based on the level of ocular primary spherical aberration (measured for a 6 mm pupil aperture) in each eye. The target spherical aberration with the contact lens fitted was set close to −0.15 µm (combined with half the value of the secondary spherical aberration and with the opposite sign), as this has been shown to maximize the eye’s depth of focus [17,18]. Using the OpTaLix-Pro^®^ 10.1 software (Optenso™, Optical Engineering Software, Igling, Germany) and following a method previously described by our research group [12], specific designs (AB+ low addition, AB− low addition, AB+ high addition, and AB− high addition) were created for clinical use. The following fitting criteria were applied:

- Eyes with negative spherical aberration: Fitting with the AB+ low addition design, switching to AB+ high addition if near visual performance was unsatisfactory.
- Eyes with positive spherical aberration < 0.5 µm (6-mm pupil): Fitting with the AB− low addition design, switching to AB+ high addition if near visual performance was unsatisfactory.
- Eyes with positive spherical aberration > 0.5 µm (6-mm pupil): Case-specific simulation and creation of a fully customized design with the appropriate central anterior surface asphericity.

Multifocal CSCL fitting was performed using biomicroscopy, fluorogram with fluorescein, and optical coherence tomography (OCT) with a Cirrus HD 500 (Carl Zeiss Meditec, Dublin, CA, USA). The distribution of the tear film below the different areas of the CSCL generated a characteristic fluorogram consisting of central tear pooling with paracentral alignment and significant edge clearing (Figure 1).

### 2.4. Post-Fitting Examination

Two follow-up visits were scheduled by the same examiner who performed the pre-fitting examinations (D.S.): 20 min and a month after lens fitting. In these check-ups, stereopsis, contrast sensitivity, defocus curve, and aberrometry were measured.

### 2.5. Data Analysis

The SPSS statistical package version 28.0.0 (IBM SPSS Inc., Chicago, IL, USA) was used to perform the statistical analysis. Non-parametric statistics were used due to the limited sample size. The Wilcoxon and Friedman tests were used for the assessment of the statistical significance of differences between the measurements of two and three consecutive visits, respectively. A *p*-value < 0.05 was considered statistically significant for the analysis.

## 3. Results

A total of 22 eyes from 11 subjects (10 female) aged between 46 and 64 years were enrolled (mean age: 53.9 ± 4.7 years). All subjects were successfully fitted with the CSCL after one or more trials and no adverse effects were reported during the 1-month follow-up. Mean flattest and steepest keratometric readings were 7.83 ± 0.33 mm and 7.72 ± 0.26 mm, respectively, with a mean keratometry value of 7.77 ± 0.29 mm. Regarding the refractive error, the mean spherical equivalent was +1.14 ± 1.75 D (range: −1.00 to +3.25 D), while the mean presbyopic addition was +2.27 ± 0.32 D (range: +1.75 to +2.75 D). The mean value of the CSCL power that was fitted was −1.20 ± 2.17 D, with an average corneal base radius of 7.26 ± 0.51 mm. All eyes were fitted with contact lenses with a diameter of 14 mm.

Table 1 shows the results of the ocular aberrometric (5 mm pupil) and stereopsis analysis before and after the contact lens fitting. Significant changes were observed in primary spherical aberration (SA), secondary spherical aberration (SA2), primary coma root mean square (RMS), and stereopsis. In contrast, no significant changes were observed in Strehl ratio and total RMS between pre- and post-fitting visits. Specifically, there was a change towards negative primary spherical aberration, whereas the secondary spherical aberration experienced a change towards a positive value. Likewise, the level of primary coma increased. Regarding stereopsis, there was an increase in the threshold that represented a trend towards a lower level of stereopsis with the CSCL fitted.

**Figure 2:**
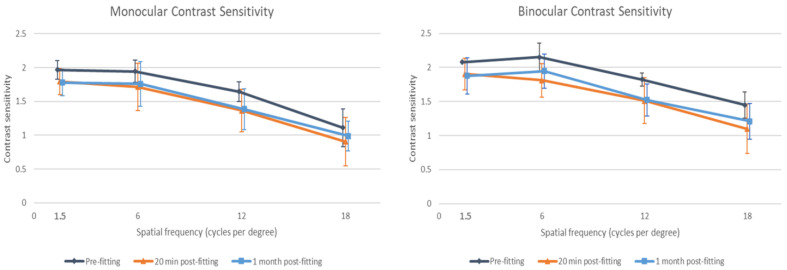
Monocular (dominant eye) and binocular contrast sensitivity before fitting, 20 min post-fitting, and 1-month post-fitting of the multifocal corneo-scleral contact lens evaluated.

Contrast sensitivity was measured binocularly and monocularly (only in the dominant eye), as shown in Figure 2. Binocularly, contrast sensitivity was significantly better with spectacle correction than with the CSCL for all spatial frequencies: 1.5 (*p* = 0.027), 6 (*p* = 0.014), 12 (*p* = 0.011), and 18 cycles/degree (*p* = 0.048). Monocularly, significant changes were found for the spatial frequencies of 1.5 (*p* = 0.027), 6 (*p* = 0.049), and 12 (*p* = 0.009) cycles/degree, but changes in monocular CS for 18 cycles/degree did not reach statistical significance (*p* = 0.109). This is shown in Figure 2.

**Figure 3:**
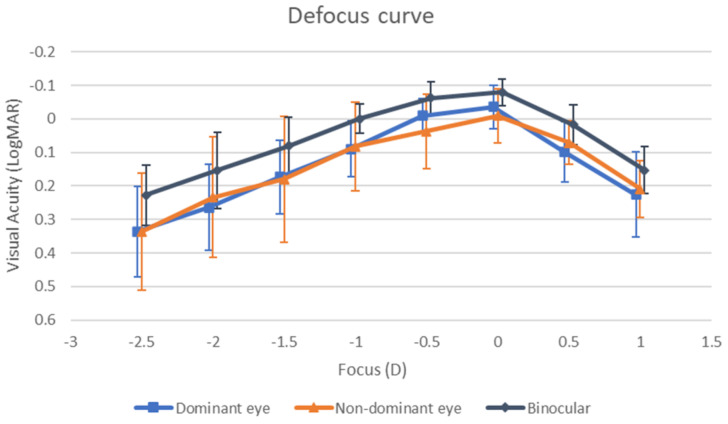
Defocus curve obtained with the multifocal corneo-scleral contact lens evaluated monocularly and binocularly.

Figure 3 shows the defocus curve of the CSCL measured under binocular and monocular conditions (both dominant and non-dominant eye). As shown, the mean corrected-distance visual acuity was 0.20 logMAR or better for defocus levels between 0.50 and −1.50 D under monocular conditions. When the visual acuity was measured binocularly, this range became wider, from +1.00 to −2.00 D.

**Figure 4:**
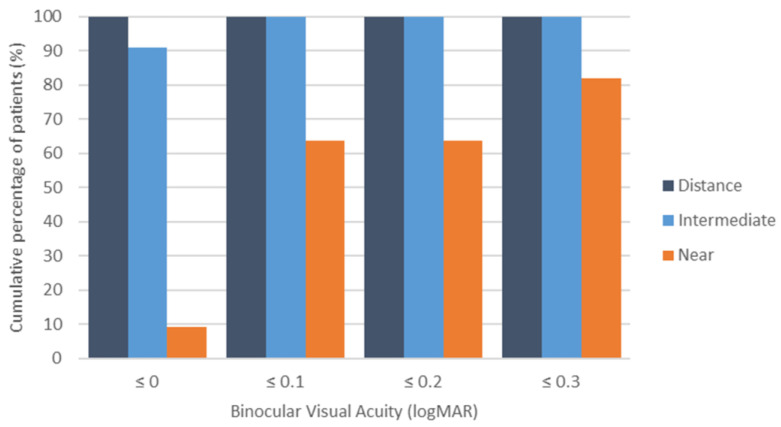
Cumulative percentage of patients with a binocular distance-corrected visual acuity equal to or better than 0.0, 0.1, 0.2, and 0.3 LogMAR for distance, intermediate, and near vision.

Finally, Figure 4 shows the distribution of corrected-distance, intermediate, and near visual acuity achieved with the CSCL fitted. As shown, all patients presented a binocular corrected-distance visual acuity equal to or better than 0 LogMAR (defocus 0.00 D), and 90.9% of patients achieved this visual acuity level at intermediate vision (defocus −1.00 D). Concerning near vision (defocus −2.00 D), 91.8% of patients presented a binocular visual acuity equal to or better than 0.3 LogMAR.

## 4. Discussion

Corneo-scleral contact lenses (CSCLs) offer several advantages for presbyopia correction. Their design creates a stable tear reservoir that simultaneously (1) reduces optical aberrations by smoothing irregular corneal surfaces and (2) maintains ocular surface hydration, particularly benefiting patients with corneal irregularities (e.g., keratoconus) or dry eye disease [19,20,21]. The mechanical stability of CSCLs provides additional optical benefits: minimal lens movement during blinking (<0.2 mm displacement) [22], reduced deformation, and the consistent induction of controlled aberrations for depth-of-focus enhancement [10]. These advantages have driven a growing interest in CSCLs for presbyopia management, though evidence remains limited. Recently, a breakthrough, customizable, multifocal CSCL (Presbycustom, Lenticon) has been introduced, featuring tear film meniscus compensation and ocular aberration customization. This study represents the first clinical evaluation of this innovative presbyopia correction approach, addressing a critical gap in the contact lens literature.

This study confirmed that the evaluated CSCL provides sufficient depth of focus to ensure functional visual acuity across various distances. Specifically, the mean binocular corrected-distance visual acuity was 0.20 logMAR or better for defocus levels between +1.00 and −2.00 D. These results are consistent with—and, in some cases, superior to—those previously reported for other soft and hybrid multifocal contact lenses [5,23,24,25,26,27,28,29]. In 2017, our research group [28] conducted a comparative study of one hybrid multifocal CL (Duette Multifocal, SynergEyes, Carlsbad, CA, USA) and two soft multifocal CLs (AirOptix from Alcon, Fort Worth, TX, USA and Biofinity from CooperVision, Pleasanton, CA, USA). The study found that the mean binocular corrected-distance visual acuity was 0.20 logMAR or better across a defocus range of +0.50 to −2.00 D for all three lenses, with no significant differences in visual acuity at any defocus level evaluated in the defocus curve. García-Lázaro et al. [29] compared the PureVision multifocal CL (Bausch & Lomb, Rochester, NY, USA) with a pinhole CL and observed no significant difference in presbyopia correction for intermediate distances (lens powers from −1.00 to −2.00 D). However, the PureVision Multifocal lens provided significantly better visual acuity at the −2.50 D vergence. In the current study, all patients achieved a binocular corrected-distance visual acuity of 0.00 logMAR or better at 0.00 D defocus, and 90.9% maintained this level at intermediate vision (−1.00 D defocus). These findings confirm the efficacy of the evaluated multifocal CL for far and intermediate distance correction. At near vision (−2.00 D defocus), 91.8% of patients exhibited a binocular visual acuity of ≤0.30 logMAR, suggesting functional near vision for most patients [30]. Future studies should explore whether near vision can be further improved with micromonovision, as demonstrated with other multifocal CL modalities [31].

This study provides the first preliminary evaluation of visual performance with a novel, multifocal CSCL. Our findings revealed a measurable reduction in stereopsis (134.7 ± 139.7 arcsec) after 20 min of lens wear, which contrasts with the better stereopsis outcomes reported by Privado-Aroco et al. for conventional (68.3 ± 33.3 arcsec) and decentered-optics multifocal scleral lenses (81.2 ± 60.9 arcsec) [9]. The literature presents conflicting evidence regarding stereopsis with multifocal corrections: while some studies of soft multifocal lenses report superior stereopsis [19,20,21], others demonstrate impairment comparable to our findings [22,32]. Notably, Privado-Aroco et al.’s pilot work also showed worse stereopsis with multifocal versus monofocal scleral lenses [8]. Several factors may explain this variability, including methodological differences, such as heterogeneity in stereopsis assessment protocols, lack of standardized testing conditions across studies, or variations in control group selection. In addition, the substantial standard deviation in our results (±139.7 arcsec) suggests considerable inter-subject variability, and the small sample size may have amplified the impact of individual outliers. For this reason, comparison between studies of multifocal CLs in terms of stereopsis should be conducted with care. This highlights the need for larger, controlled studies with standardized protocols, with direct comparisons between different multifocal designs under matched conditions.

Although some high-order aberrations (coma, SA, SA2) increased after the fitting of the CSCL evaluated, the Strehl ratio and total ocular RMS did not experience significant changes. These changes in SA and SA2 were mainly induced by the lens design, which combines SA and SA2 with opposite signs, leading to an increase in the depth of focus [16,17]. Additionally, as the CSCL evaluated does not have peripheral toricity, some levels of decentration that were mainly due to the naso-temporal scleral asymmetry of the human eye may have been present, contributing potentially to the observed increase in coma [10,33]. In any case, the levels of SA, SA2, and primary coma found with the multifocal CSCL fitted were within the range of normality [34], with no abnormal inductions of high-order aberrations. It should also be noted here that the adjustment of the toricity of the landing zone of an SCL could be beneficial in reducing lens flexure and rotation [35], leading to an improvement in the level of neutralization of high-order aberrations.

As in most previous studies evaluating the impact of multifocal CLs in CS [8,9,36,37,38], a trend towards a reduction in contrast sensitivity was found when assessed with the multifocal CSCL compared to spectacle correction. However, compared to the range of normality defined for most of the CS tests that are available, the level of CS obtained with the CSCL evaluated was within the range of normality across all spatial frequencies, despite its reduction [39]. Therefore, the expansion of the depth of focus with the CSCL evaluated was generated by inducing a controlled increase of high-order aberrations, maintaining the levels of visual quality within an acceptable level. Indeed, as previously mentioned, the defocus curve showed that the CSCL evaluated enabled good visual performance at far and intermediate vision, with functional results in near vision [30]. The defocus curve presented a smooth profile, characterized by a gradual decrease in near vision and a peak in distance vision, indicating an effective distribution of focal power across distances.

This was a pilot study and, therefore, has several limitations. First, the small sample size may limit the generalizability of the results to a broader population, but the aim of the current study was to confirm the clinical viability of this novel CSCL. Further studies with larger sample sizes—including a more balanced sex distribution—are needed to analyze clinical outcomes across subgroups (e.g., refractive error, pupillometry, age). Moreover, the lack of a control group using multifocal soft CL or CSCL limits the ability to directly compare the performance of the CSCL against current options. However, it should be considered that this is a pilot study to confirm the viability of this type of CL, and a new, randomized, controlled clinical trial has been designed (including a control group) to confirm exactly the real benefit of the new CSCL over a commercially available multifocal soft CL. Another limitation is the potential decentration due to the lack of toricity in the peripheral area of the lens, which may have significantly impacted the visual quality. This limitation should be overcome in future modifications of the design of the CL.

## 5. Conclusions

The CSCL Presbycustom effectively restores visual acuity across distances in presbyopic patients while maintaining functional visual quality and stereopsis. Further research with larger sample sizes is needed to validate these preliminary findings and assess its comparative efficacy relative to existing multifocal contact lenses for presbyopia.

## Figures and Tables

**Table 1:** Aberrometry (5 mm pupil) and stereopsis measurements pre-fitting and 20 min post-fitting. Abbreviations: SD, standard deviation; RMS, root mean square; SA, primary spherical aberration; SA2, secondary spherical aberration.

	Pre-Fitting		Post-Fitting		
	Mean	SD	Mean	SD	*p*-Value
Total RMS (µm)	1.90	1.77	3.16	2.13	0.182
Strehl ratio	0.020	0.027	0.018	0.018	0.965
SA (µm)	0.0127	0.103	−0.296	0.297	0.033
SA2 (µm)	−0.008	0.21	0.081	0.78	0.009
Coma RMS (µm)	0.173	0.130	0.882	0.557	0.008
Stereopsis (arc sec)	61.1	64.1	134.7	139.7	0.005

## Data Availability

Data are available upon reasonable request to the authors.

## Abbreviations

The following abbreviations are used in this manuscript:

CSCL	Corneo-scleral contact lens
CS	Contrast sensitivity
IOL	Intraocular lens
SCL	Scleral contact lens
CL	Contact lens
SA	Primary spherical aberration
SA2	Secondary spherical aberration
RMS	Root mean square
